# Continuous positive airway pressure versus usual care for obstructive sleep apnoea in pregnancy: a two-step pilot trial

**DOI:** 10.1186/s40814-025-01645-1

**Published:** 2025-06-05

**Authors:** Rachael Nugent, Caroline de Costa, Leonie Callaway, Shiv Erigadoo, Elise Gilbertson, Chris Brown, Joanna Perry-Keene, Rebekah Shakhovskoy, Lauren Kearney

**Affiliations:** 1 Sunshine Coast Hospital and Health Service, Birtinya, QLD 4575 Australia; 2 University of Queensland, Brisbane, Australia; 3 James Cook University, Townsville, Australia; 4 Royal Brisbane and Women’s Hospital, Brisbane, Australia; 5 University of the Sunshine Coast, Buderim, Australia; 6 Griffith University, Sunshine Coast, Australia

**Keywords:** Pregnancy, Continuous positive airway pressure, Obstructive sleep apnoea, Obesity

## Abstract

**Background:**

Obstructive sleep apnoea (OSA) is associated with increasing body mass index (BMI) and affects up to 15% of pregnant women. OSA in pregnancy can be associated with adverse maternal and neonatal outcomes. Continuous positive airway pressure (CPAP) may be an effective treatment for OSA during pregnancy.

**Aim:**

To examine the feasibility and acceptability of screening women with a BMI ≥ 35 kg/m^2^ for OSA in pregnancy, followed by randomisation to treatment with CPAP or no CPAP for women diagnosed with moderate to severe OSA.

**Methods:**

This study was a single-centre, two-stage pilot study. Firstly, all consenting participants were screened for OSA and then, only if diagnosed with moderate to severe OSA (*AHI* ≥ 15), randomised to CPAP or no CPAP. The feasibility of the OSA screening was determined by recruitment rates. A priori criteria of > 20% recruitment to the sleep study were considered feasible. The acceptability of the sleep study was examined using the completion rate and reasons for withdrawal prior to the sleep study. The acceptability of CPAP randomisation was assessed by completion rates for women randomised, adherence to CPAP, reasons for withdrawal, and the review of quality-of-life measures over the course of pregnancy.

**Results:**

Ninety-six women with a BMI ≥ 35 kg/m^2^ before 26 completed weeks of pregnancy were invited to participate from the Sunshine Coast University Hospital and Health Service. Overall, 36 women enrolled, giving a recruitment rate of 37%. A total of 75% (*n* = 26) of recruited women completed OSA screening (clinical history, questionnaires, and formal sleep study). Subsequently, six (6%) of invited women had an apnoea hypopnoea index (AHI) ≥ 15/h and were diagnosed with moderate to severe OSA and underwent randomisation to receive treatment with CPAP (*n* = 4) or no CPAP (*n* = 1) until birth. One woman withdrew after randomisation to treatment. Nine women withdrew prior to full completion of OSA screening and one following an inconclusive sleep study. Women who had a sleep study found it acceptable, and in women randomised to CPAP, adherence averaged 4.86 h/night. Quality-of-life measures were similar when measured over two time points in pregnancy.

**Discussion:**

A definitive trial screening women for OSA and subsequent randomisation to treatment or no treatment may be feasible. Future trials should be resourced to expand inclusion criteria, improve accessibility for participants, evaluate clinical and cost-effectiveness, and investigate partial treatment effects.

**Trial registration:**

ACTRN, ACTRN12621001523897. Registered 9 September 2021 — retrospectively registered, https://www.anzctr.org.au/Trial/Update/Step8_Update.aspx?id=382640.

## Key messages regarding feasibility

• What uncertainties exist regarding the feasibility?

Key feasibility issues were the ability to recruit effectively for the sleep study diagnostic test and CPAP intervention and acceptability of both the sleep study and the intervention in pregnancy.

• What are the key feasibility findings?

Screening for OSA in pregnancy is both feasible and acceptable during pregnancy. Randomisation for treatment with CPAP versus no treatment is acceptable. Alterations need to be made to the inclusion criteria and study design to improve the feasibility of randomising women with OSA in pregnancy to CPAP or usual care.

• What are the implications of the feasibility finding for the design of the main study? Adequate resource allocation to facilitate comprehensive screening and recruitment is pivotal to the success of a trial. Utilisation of more accessible home-based diagnostic modalities may improve recruitment. Broadening inclusion criteria to include gestational age up to 34 weeks, and randomising women diagnosed with mild-moderate OSA, should be considered.

## Introduction

Obstructive sleep apnoea (OSA) is a common disorder characterised by repetitive episodes of nocturnal breathing cessation due to upper airway collapse, resulting in decreased oxygen saturation [1]. OSA in pregnancy has an estimated prevalence of approximately 15% of pregnancies in the second and third trimesters [2]. OSA is associated with increased rates of pregnancy complications such as hypertensive disease and pre-eclampsia, gestational diabetes, and caesarean birth [3–6]. It is also associated with an increased risk of severe maternal morbidity such as cardiomyopathy, hysterectomy, and intensive care unit (ICU) admission [3]. For the infant, OSA has been associated with preterm birth [2], lower birthweight [2, 4, 6], and higher rates of neonatal intensive care unit (NICU) admission [2, 3, 5, 6]. Rates of OSA in pregnancy rise with increasing BMI [3, 7, 8], chronic hypertension [3, 5], and diabetes [3].

Continuous positive airway pressure (CPAP) to treat OSA potentially acts through multiple pathophysiological pathways leading to a reduction in the incidence and severity of hypertensive disease in pregnancy. Intermittent hypoxaemia caused by OSA may be an upstream mediator of placental disease by inducing inflammation, sympathetic nervous system activation, oxidative stress, and endothelial dysfunction [9]. The treatment of OSA with CPAP may reduce intermittent hypoxaemia, modifying several pathways at all gestations. A systematic review examining the effectiveness of CPAP for OSA in pregnancy suggested that maternal and foetal outcomes in women with OSA may be modified by CPAP treatment [10]. CPAP in pregnancy may be associated with a reduction in blood pressure and pre-eclampsia, a reduction in preterm birth, and an increase in mean birthweight [10]. However, uncertainty remains in concluding efficacy for CPAP as a treatment for OSA in pregnancy due to the small study sizes and limited ability to test clinically significant outcomes [10].

Systematic review evidence suggests CPAP in pregnancy is well tolerated with reasonable adherence [10], although adherence as low as 2% has been reported [11]. The largest randomised controlled trial (RCT) of 340 high-risk women in Thailand reported CPAP adherence rates of 32.7% [12]. Women in this study treated with CPAP from the first trimester experienced a reduction in hypertensive disorders, particularly pre-eclampsia [12]. A reduction in diastolic blood pressure in this study appeared to be directly proportional to time spent on CPAP [12], suggesting that some therapeutic effect may be possible even with reduced or inconsistent use. The average time on CPAP was 2.5 h per night [12]. One non-randomised study using a CPAP treatment window of only 4 weeks in pregnancy demonstrated a reduction in severe forms of hypertensive syndrome during pregnancy [13]. The dose–response relationship for CPAP in pregnancy requires further delineation.

The prevalence and severity of obstructive sleep apnoea worsen throughout pregnancy [14] and are more common in women with higher BMIs. Although the diagnosis and treatment of OSA in pregnancy are recommended by international guidelines for women with higher BMIs [15], there is limited data to guide this practice, including which screening instruments to use, how feasible this screening is to undertake in busy maternity units, and the acceptability of this screening to women. Furthermore, RCT data on CPAP effectiveness during pregnancy is minimal, especially from high-resource settings, although a multicentre RCT currently recruiting in the United States of America (USA) is testing the utility of CPAP for the treatment of OSA in pregnancy [16]. Limited high-quality trial evidence is common within women’s health generally, and even more so for pregnant women who are often excluded due to safety concerns, creating an inequitable evidence gap [17].

Investigation in the Australian context has not occurred to date and is warranted due to population differences with other settings, such as increasing average maternal BMI [18]. Australian studies have historically focused on lifestyle modifications to address comorbidities associated with raised BMI in pregnancy, with mixed results [19]. Concern regarding the long-term efficacy of these studies has also been recently questioned when targeting factors such as gestational weight gain alone [20]. Arguably, this prompts researchers and clinicians to explore alternative and novel interventions to address this. Obstructive sleep apnoea in pregnancy is a potentially modifiable risk factor for women with higher BMIs, and its diagnosis and treatment may significantly improve outcomes. Currently, in Australia, there is not a universal approach to screening, diagnosing, or treating OSA during pregnancy. The feasibility of OSA screening and the acceptability of this as a treatment intervention to pregnant women are important to understand.

Therefore, this study aimed to address this gap within a two-step pilot trial, aiming to evaluate the feasibility and acceptability of (i) screening pregnant women with BMI ≥ 35 kg/m^2^ for OSA using an at-home or in-hospital sleep study and (ii) randomising pregnant women diagnosed by the sleep study with moderate to severe OSA (*AHI* ≥ 15) in the second trimester of pregnancy to treatment with CPAP or no treatment (standard care).

## Methods

A two-step pilot trial was undertaken, with all consenting women screened firstly for OSA in pregnancy, and then, if diagnosed, a randomised allocation to CPAP or standard care occurred. Following enrolment by a study investigator, baseline sleep study questionnaire and quality of life (QoL) data were recorded.

### Screening stage

All participants were invited to complete full OSA screening: clinical history, questionnaire completion (see the “Data collection”), and a formal sleep study in the second trimester at home or in hospital. Diagnosis of OSA was defined, following the screening, if participants scored a AHI ≥ 15.

#### Feasibility measures

Progression criteria for feasibility of the screening component included a recruitment rate > 20% of eligible women or 80 patients for the sleep study over the study period and were set a priori [21]. A total of 20% is at the lower end of recruitment rates [22, 23] for feasibility, but this was tolerated due to two factors. Firstly, the 50% long-term compliance rate with CPAP treatment in the general population [24] was considered a potential limiting factor in recruitment. Secondly, BMI ≥ 35 kg/m^2^ is common, affecting an estimated 7% of the pregnant population, therefore offering a large potential sample and high overall recruitment numbers. The recruitment rate for the screening stage was the proportion of women recruited of those who were invited. Acceptability of the sleep study was assessed by reviewing adherence data, completion rates, and reasons for withdrawal. The completion rate for the screening test was the proportion of women who completed the sleep study of those who were recruited.

### Pilot randomised controlled trial stage

Following the sleep study, participants with an AHI ≥ 15 (moderate to severe OSA) were randomised to CPAP or usual care. Women with *AHI* 0–15, therefore mild OSA, did not progress to the randomisation stage, as treatment for OSA was not recommended within this subset. This was informed by the already existing evidence that mild OSA is not associated with adverse health outcomes [25], and they therefore continued to receive usual antenatal care. Participants with *AHI* ≥ 15 underwent block randomisation in groups of 10 undertaken by an unblinded investigator using a computer-generated random number list. Women in this group were randomised to routine antenatal care as per local guidelines [26, 27] or routine care with CPAP using an auto-titrating CPAP device. CPAP treatment included comprehensive fitting and advice regarding the use of the device, including collection of adherence data related to the length of treatment collected directly from the machine. QoL questionnaires were repeated at 36- to 38-week gestation for all participants, and histopathological examination of the placenta was undertaken following birth.

Participants and research staff were unblinded following allocation, due to the nature of the intervention. Feasibility and acceptability outcome assessment was undertaken unblinded by the investigators. Pathologists completing placental histopathological assessment were blinded to the participants’ status with regard to the intervention (CPAP or no CPAP).

#### Feasibility measures

The recruitment rate to the randomised intervention was calculated as the proportion of women who underwent randomisation of those who were invited to the initial study. Acceptability of the sleep study and CPAP randomisation were assessed by reviewing completion rates, reasons for withdrawal, adherence to CPAP, and quality-of-life measures. The completion rate for the randomised intervention is reported as the proportion of women completing the protocol of those who were invited to. Adherence to CPAP (measured in hours/day) was considered a proxy measure of acceptability of the intervention and was calculated as a mean for women randomised to CPAP. A priori progression criteria for adherence were ‘therapeutic adherence’ or > 5 h/day across the trial, more than 70% of the time [21].

#### Setting

This was a single-site study, conducted in a large regional hospital in South-East Queensland, Australia. Annually, 3300 births occur in this hospital.

#### Participants and sampling

Inclusion criteria for the overall study were as follows: BMI ≥ 35 kg/m^2^ and singleton pregnancy at < 26-week gestational age. Exclusion criteria were as follows: high-risk first trimester screen including noninvasive prenatal testing or foetal anomalies [28], multiple gestation, known sleep-disordered breathing with mechanical therapy, coronary artery disease, congestive heart failure, or cardiomyopathy. Recruitment occurred in the maternity outpatient unit where client health records were screened, and eligible women were invited to participate by their reviewing clinician. Women who expressed interest were contacted by a member of the research team. Following discussion, the outcome of the research team contact was recorded as follows: unable to contact; declined, including reason why; or agreed to be recruited. Written consent was undertaken by named investigators from the research team, face to face or via telehealth.

#### Sample size justification

Estimated participant numbers were based on current birth rates at the recruitment site [29] and an estimated proportion of women with a BMI ≥ 35 kg/m^2^ of approximately 7%. Recruitment of 20% of eligible women would represent 40 women in a year, satisfying our a priori feasibility criteria. The sample size was estimated to be 80 women for a 2-year recruitment period, the initial proposed sampling timeframe. Pilot and feasibility studies often require between 12 and 35 patients per arm [30, 31]. It was estimated that recruitment of 80 participants to the sleep study would facilitate recruitment of at least 12 participants to the intervention arm of the study. In addition, a sample size of 80 was considered large enough to generate a reasonably precise prevalence estimate for OSA in the study population. The proportion of women affected by moderate to severe OSA (*AHI* ≥ 15) was estimated to be 30% [32]. The sample size required to estimate a prevalence with a margin of error of 10 percentage points with 95% confidence was calculated to be 81 [33]. This sample size was also considered adequate to facilitate review of the integrity of the study protocol.

Initial planned recruitment was from February 2020 to February 2022; however, the commencement of recruitment was delayed to October 2020 due to the COVID- 19 pandemic. The study protocol was adjusted to extend the recruitment window to a 3-year period, closing in October 2023, and to extend the gestational age for recruitment to 26 weeks. Both were in response to low overall recruitment.

#### Data collection

Following recruitment, baseline data collection was undertaken by a research midwife. Participant characteristics collected included age, gestation, parity, country of birth, Aboriginal or Torres Strait Islander status, BMI, smoking, and alcohol consumption in pregnancy. Relevant maternal medical and pregnancy history was collected by questionnaire including history of essential hypertension, diabetes, thyroid disease, renal disease, liver disease, gestational hypertension, pre-eclampsia, eclampsia, preterm labour, or placental abruption. The STOP-Bang [34], Epworth Sleepiness Score [35], and Berlin sleep score [36] were recorded at recruitment, over the phone or face to face, or at the time of sleep study. Modified PROMIS Global Short Form [37] QoL questionnaires were collected by email, phone, or research nurse phone call at recruitment and 36–38 weeks.

Clinical outcome data was collected by client health record review and included a composite primary outcome of gestational hypertension, pre-eclampsia, eclampsia, low birthweight/small for gestational age, preterm birth less than 37 weeks, and stillbirth. Other outcomes collected via client health record review included gestational weight gain, gestational diabetes, onset of labour, mode of birth (vaginal birth, vacuum, forceps, elective or emergency caesarean section), estimated blood loss, blood transfusion, ICU admission, and maternal death. Outcomes of gestational hypertension, pre-eclampsia, gestational diabetes, and low birthweight or small for gestational age are reported as per local guidelines [38, 39]. Neonatal outcome measures included neonatal birthweight, gestation at birth, admission of neonate to the neonatal unit, neonatal length of hospital stay, neonatal death, and neonatal respiratory support.

#### Data analysis

Data was entered into a secure, password-protected web-based electronic case-report format and coded for patient confidentiality within a password-protected file. Descriptive statistics only are presented as there were inadequate participants for meaningful group comparison. Binomial data is described as numbers and percentages. Continuous variables are described as mean and standard deviation or medians with interquartile ranges. Groups compared were *AHI* < 5, *AHI* 5–14.99 (non-randomised), and *AHI* ≥ 15 who underwent randomisation for the CPAP intervention. Questionnaire results were entered into a purpose-built Microsoft Excel database for data cleaning and for statistical analysis.

Human research ethics approval was granted by the Prince Charles Hospital Human Research and Ethics Committee (HREC/2019/QPCH/50233) and the University of Queensland (2022/HE002216). The protocol was registered with the Australian and New Zealand Clinical Trials Registry (ACTRN12621001523897).

## Results

**Fig. 1:**
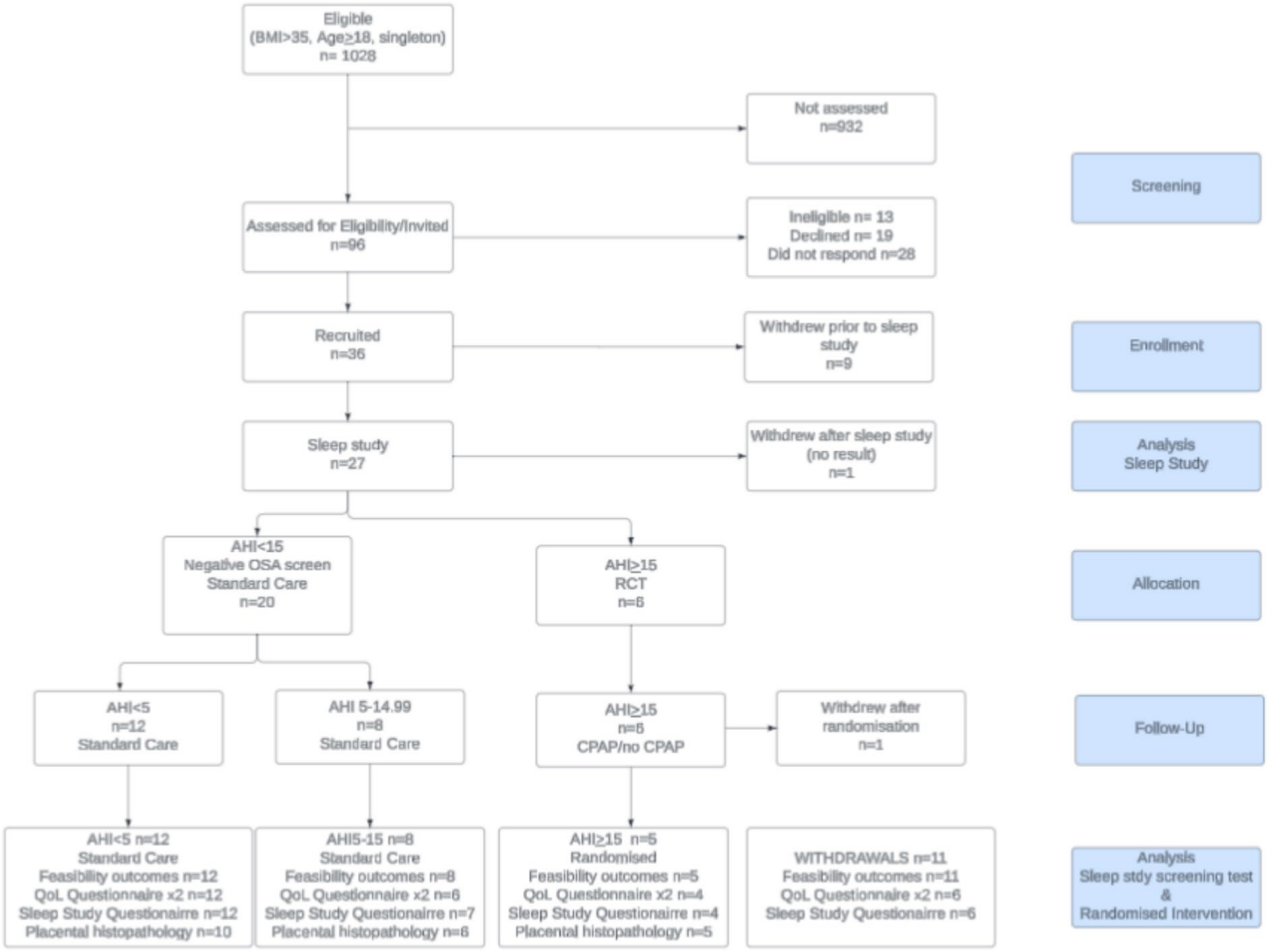
CONSORT diagram

In the study timeframe, 1028 women met eligibility criteria (see Fig. 1). A detailed screening log revealed that 15 women were ineligible to participate owing to high-risk first trimester screening results, moving their care to another facility, gestation at or near 24–26 weeks, BMI < 35 kg/m^2^, or previous diagnosis with OSA or cardiomyopathy. Twenty-seven women who were invited to participate and consented to being contacted by the research team did not respond when contacted; 17 women declined participation owing to work and family commitments or psychological stressors.

Baseline data for all participants is outlined in Table 1, grouped by AHI. At baseline, demographic data including age, BMI, parity, and history of complications in previous pregnancies or current medical conditions were recorded via questionnaire. Figure 1 outlines the number of participants completing sleep questionnaires, QoL questionnaires, and the number of placentas assessed and reported. Placental histopathology results are reported elsewhere.

**Table 1:** Baseline characteristics of the participants recruited for stage 1 screening

	Not randomised Routine antenatal care		Randomised Routine care +/− CPAP	Total
Characteristics	*AHI* < 5 No OSA *N* = 12	*AHI* 5–14.99 Mild OSA *N* = 8	*AHI* ≥ 15 Moderate-severe OSA *N* = 5	Total *N* = 25
Maternal age in years (mean, SD)	28.8 (5.3)	30 (3.9)	32.2 (1.6)	29.8 (4.4)
Nulliparous, no. (%)	9 (75)	5 (63)	3 (60)	17 (68)
Gestation at sleep study in weeks* (mean, SD)	24 + 5 weeks (4 weeks)	26 + 1 (4.1 weeks)	22 + 5 (2.5 weeks)	24 + 6 (3.9 days)
Average BMI in kg/m^2^ (mean, SD)	42.3 (5.8)	41.7 (6.8)	47.8 (7.7)	43.3 (6.7)
Aboriginal and/or Torres Strait Islander	0 (0)	1 (13)	0 (0)	1 (4)
Country of birth - Australia - New Zealand - India - UK	10 (83) 2 (17) 0 (0) 0 (0)	7 (88) 0 (0) 1 (12) 0 (0)	4 (80) 0 (0) 0 (0) 1 (20)	21 (84) 2 (8) 1 (4) 1 (4)
Prior pregnancy is affected by the following: Pre-eclampsia, eclampsia, growth restriction, gestational hypertension, preterm labour, and placental abruption	0 (0)	0 (0)	0 (0)	0 (0)
History of preterm labour	0 (0)	0 (0)	0 (0)	0 (0)
Neck circumference (cm) mean, SD	38.6 (2.3)	38.1 (2.6)	41.3 (4.6)	39 (3)
Smoking	0 (0)	1 (13)	1 (20)	2 (8)
Alcohol consumption	0 (0)	0 (0)	0 (0)	0 (0)
Chronic hypertension	2 (17)	0 (0)	0 (0)	2 (8)
Type II diabetes	0 (0)	0 (0)	0 (0)	0 (0)
Hypothyroid	1 (8)	0 (0)	1 (20)	2 (8)
Liver disease	0 (0)	0 (0)	2 (40)	2 (8)

^*^Gestational age is reported as “X + Y”, where X is the number of weeks and Y is the additional days. For example, “24 + 5” means 24 weeks and 5 days

Maternal and neonatal outcome data for women recruited to the sleep study is reported in Table 2, grouped by AHI. AHI group signifies the severity of OSA, with *AHI* < 5 no OSA, 5–14.99 mild OSA, and *AHI* ≥ 15 moderate to severe OSA. Numbers were too small to detect any significant differences or associations between the groups.

**Table 2:** Pregnancy outcomes in women recruited for stage 1 screening

	Not randomised Routine care		Randomised Routine care +/− CPAP	TOTAL
	*AHI* < 5 No OSA *N* = 12	*AHI* 5–14.99 Mild OSA *n* = 8	*AHI* ≥ 15 Moderate-severe OSA *n* = 5	Total *n* = 25
Composite clinical outcome gestational hypertension, pre-eclampsia, eclampsia, low birthweight/small for gestational age, preterm birth less than 37 weeks, stillbirth﻿^#^ (*n*, %)	1 (8)	1 (13)	2 (40)	4 (16)
Gestational hypertension (*n*, %)	2 (17)	0 (0)	1 (0)	3 (12)
Preterm birth (< 37 weeks) (*n*, %)	0 (0)	0 (0)	1 (20)	1 (4)
Foetal growth restriction < 5 th percentile (*n*, %)	1 (8)	0 (0)	0 (0)	1 (4)
Large for gestational age > 90 th percentile (*n*, %)	2 (17)	0 (0)	1 (20)	3 (12)
Gestational diabetes (*n*, %)	4 (33)	3 (38)	2 (40)	9 (36)
Onset of labour - No labour — CS (*n*, %) - Induced (*n*, %) - Spontaneous (*n*, %)	4 (33) 6 (50) 2 (17)	3 (38) 4 (50) 1 (13)	1 (20) 3 (60) 1 (20)	8 (32) 13 (52) 4 (16)
Mode of birth - SVB (*n*, %) - Instrumental (ventouse or forceps) (*n*, %) - Elective CS (*n*, %) - Emergent CS (*n*, %)	5 (42) 1 (8) 3 (25) 3 (25)	3 (38) 1 (13) 1 (50) 3 (38)	3 (60) 0 (0) 1 (20) 1 (20)	11 (44) 2 (8) 5 (20) 7 (28)
• Estimated blood loss (EBL) (mean, SD) in mL • Blood transfusion (*n*, %) • Maternal ICU admission (*n*, %)	645 (441) 0 (0) 0 (0)	525 (306) 0 (0) (0)	490 (472) 0 (0) 0 (0)	575 (398) 0 (0) 0 (0)
Neonatal outcomes				
Admission to NNU (*n*, %)	3 (25)	3 (38)	1 (20)	7 (28)
Neonatal length of stay (median, IQR) in days	3 [1.8–3.3]	3 [2, 3]	2 [2, 3]	3 (2, 3)
Neonatal respiratory support (*n*, %)	1 (13)	1 (13)	0 (0)	2 (8)
Gestational age at birth in weeks (mean, SD)	39 + 1 (1.0)	39 + 2 (1.0)	37 + 6 (1.8)	39 + 0 (1.3)
Average birthweight in grams (mean, SD)	3348 (573)	3241 (223)	3470 (735)	3338 (512)

^#^No maternal or neonatal deaths or stillbirth recorded

QoL data is reported in Table 3. The scores represent the results of the QoL questionnaire, the Modified PROMIS Global Short Form [37, 40], in which higher component score values represent higher quality of life. QoL for mental and physical health scores is displayed according to AHI group.

**Table 3:** Revised mental health (MH-PP) and physical health (PH-PP) scores at recruitment and at 36–38 weeks

	*AHI* < 5 *N* = 12	*AHI* 5–14.99 *N* = 7	*AHI* ≥ 15 *N* = 4
Mental health (MH-PP) (mean, SD)			
Pregnancy baseline (recruitment)	3.32 (0.7)	3.32 (0.86)	2.9 (0.6)
Pregnancy 36–38 weeks	3.35 (0.46)	3.54 (0.89)^a^	3.2 (0.69)
Physical health (PH-PP)			
Pregnancy baseline (recruitment)	3.22 (0.57)	2.71 (0.64)	3.69 (0.43)
Pregnancy 36–38 weeks	3.37 (0.51)^b^	2.70 (0.79)^a^	3.75 (0.35)

*AHI* < 5, no OSA (usual care); *AHI5 - 15*, mild OSA (usual care); *AHI* ≥ 15, moderate-severe OSA (invited to randomisation)

^a^*N* = 6, one participant completed only the recruitment QoL questionnaire

^b^One participant answered four of the five questions regarding physical health — the average of these was taken

### Feasibility outcomes — screening study and randomised intervention

From 96 women invited to participate, 36 (37.5%) were recruited for the stage 1 screening study. Six of the 96 women were qualified for the stage 2 randomisation (6.3%). Had OSA been defined as *AHI* ≥ 5 as in other international trials, 14.5% of women approached would have been eligible for randomisation. Overall, low numbers of women were recruited to participate, with only 9.6% of the eligible population screened for participation.

### Acceptability outcomes — screening study

Of the 36 women recruited, 27 (72%) women completed the sleep study. Of the nine who withdrew prior to conducting a sleep study, cited reasons were as follows: work and family commitments, ‘life was too busy’, or the additional appointment load was undesirable.

One woman withdrew following the sleep study (prior to randomisation) after being unable to tolerate a home sleep study and declined further participation.

### Acceptability outcomes — randomised intervention

Five women (83%) undergoing randomisation completed the study per protocol. Of the six women diagnosed with an *AHI* ≥ 15, all were randomised; however, one woman (with AHI of 26) who was randomised to CPAP treatment withdrew, stating she would be unable to tolerate the device owing to a concurrent COVID- 19 infection.

Adherence data was collected directly from the CPAP machine for women allocated to treatment and used as a proxy measure of acceptability. Women allocated to CPAP treatment in pregnancy had varying adherence to the device. Usage ranged from 0.65 h per day to 7.2 h per day, with average usage for the study of 4.86 h per night. Retention rates to postpartum follow-up were 60%, with three women in the randomised group attending for postpartum sleep team review.

## Discussion

We report the first randomised data for the treatment of OSA during pregnancy in an Australian population, contributing to a small pool of international randomised data [12, 14, 41, 42]. Our results provide foundational information for a definitive RCT and identify aspects of the protocol which need revision to provide quality data, supported by adequate recruitment.

### Feasibility of screening for OSA with a sleep study

The trial met its recruitment rate progression criteria [21] by recruiting 37.5% of women approached. Although the trial recruited one participant per month, which is average for RCTs [23], absolute recruitment fell below expectation because only 10% of eligible women were approached for participation (Fig. 1). Though progression criteria were theoretically met, overall recruitment fell well below the expected recruitment of 80 women*.* Our project design estimated that nearly all eligible women would be invited to participate in the study; however, this was not achievable with the allocated resources. Other contributing factors include the COVID- 19 pandemic response which increased maternity services delivered via telehealth as a social distancing policy [43]. Many potential participants’ initial hospital reviews were ‘too close to 24–26 weeks’ to allow recruitment. Challenges recruiting in obstetric trials [44] and trials involving CPAP [11, 12] have been previously reported. Medical staff have voiced feeling ‘pressured’ and ‘burdened’ by the additional task of recruitment [45]. Pregnant women report the primary barrier to participation in clinical research as inconvenience [46]. A significant increase in the screening of the eligible population is required to make a larger study feasible [21].

Increasing screening rates may be achievable via the adoption of active recruitment strategies and by providing adequate obstetrician and midwife support [47]. Funding a research midwife for recruitment, screening, and consenting eligible women would increase recruitment. Active recruitment strategies foster clinicians meeting directly with patients and discussing the study in person [47]. Expanding recruitment strategies to incorporate social media and snowball sampling may also enhance recruitment [48]. Including pregnant and postnatal women in the early stages of research design may increase uptake [49]. Broadening inclusion criteria to high-risk conditions such as a history of growth restriction or hypertensive disease in pregnancy would increase the available population for recruitment. Broadening the recruitment criteria further to encompass all pregnancies would boost recruitment but may reduce the diagnostic yield of a sleep study. Increasing the gestational age limit to 34 weeks would also boost overall recruitment, although the optimal timing of initiation and duration of CPAP treatment in pregnancy is unclear.

The average gestation of women undertaking a sleep study in our cohort was 25 weeks. While the therapeutic window for CPAP may be enhanced in the first trimester owing to the potential for reduced hypoxic events to modify trophoblast invasion, several proposed therapeutic mechanisms underpinning CPAP treatment for the reduction of placental disease persist into the second and third trimesters [9]. Treatment introduced later in pregnancy may modify pathways associated with impaired trophoblast invasion, placental hypoxia, endothelial dysfunction resulting from the imbalance of angiogenic factors and oxidative stress, and reduce systemic inflammation [9, 50]. Screening for OSA in the second and third trimesters may increase the diagnostic yield of the sleep study but would result in shorter windows of treatment. The impact of gestation at initiation and duration of treatment on outcomes should be elicited in future studies.

### Acceptability of the sleep study

A total of 75% of women recruited completed a sleep study at home (*n* = 2) or in hospital (*n* = 25). Thus, a sleep study was mostly acceptable to women. Feasibility studies in pregnant women typically describe retention or completion rates of 80–90% [51, 52]. The majority of women withdrawing (*n* = 9) from the research did so before the sleep study for personal reasons unrelated to the study itself. This was despite an in-home sleep study being offered as an alternative to a hospital-based sleep study, a practice with demonstrated feasibility [53]. The inconvenience of research is a known factor for women withdrawing from RCTs [46]. Appointment burden borne by women, represented by both routine and complex care requirements, is an example of the care work ‘disproportionately performed by women’ [54]. Utilising electronic data collection instruments, e-consents, and optimisation of at-home technologies may overcome some of these barriers [53]. Maternity researchers must address this sex disparity in unpaid care work as much as possible by reducing appointment and survey burden and improving flexibility and accessibility to research participation for women in pregnancy. Participation incentives could be given, though this approach has had mixed results [55].

The acceptability of the intervention was assessed via QoL questionnaires provided at two separate time points [21]. In person and phone collection of survey data resulted in higher completion rates than email surveys [56]. Completion rates for both questionnaires were 88%, with no decrease in mental or physical QoL measures for any group participating in the study.

### Feasibility of CPAP as a randomised intervention

The recruitment rate to the randomised intervention (CPAP or no CPAP) of women invited was low at 16.7%. This was expected as screening for OSA prior to randomisation is a necessary component of the trial protocol. Lowering the threshold for randomisation to women with an *AHI* ≥ 5 would improve overall recruitment and bring the protocol in line with international trials [12–14]. Future trials should assess treatment in women with an AHI 5–30. Women with severe OSA or *AHI* ≥ 30 should be directed to CPAP treatment and postpartum follow-up, given RCT data suggesting that the benefit of treating severe OSA in pregnancy likely outweighs the risk [57].

### Acceptability of the intervention

The completion rate for women allocated to the randomised arm of the study was 83%. One woman (of six total) withdrew after being allocated to the intervention arm, stating she was unable to tolerate the device due to COVID- 19 comorbidity. Adherence to the intervention was varied in the small group of women allocated with an average of it. The a priori progression criteria for adherence were ‘therapeutic adherence’ or > 5 h/day across the trial, more than 70% of the time [21]; however, these progression criteria warrant review. A dose–response relationship may exist for CPAP for OSA in pregnancy, making partial treatment potentially beneficial [12]. Further exploration of the dose–response relationship between CPAP and hypertensive disease in pregnancy is warranted.

### Composite clinical outcome

The composite outcome measure (any one of gestational hypertension, pre-eclampsia, eclampsia, low birthweight/small for gestational age, preterm birth less than 37 weeks, stillbirth) occurred in 24% of participants. No stillbirths were recorded. The sample size was too small for a prevalence calculation for this composite outcome. Since the commencement of our feasibility study, several larger trials have demonstrated a reduction in hypertensive disease in pregnancy in women with OSA diagnosed and treated from the first trimester of pregnancy [12, 13, 41]. Further investigation of the impact of CPAP on hypertensive disease should focus on the impact of adherence, duration, and gestation of commenced therapy.

### Strengths and limitations

Our study was limited overall by relatively low total recruitment numbers and low retention rates to postnatal follow-up with only 3 of 5 (60%) women diagnosed and treated for moderate or severe OSA completing a postpartum sleep review. A more focused effort to follow up women after evidence of OSA in pregnancy is required, given that a diagnosis of OSA can be associated with increased lifetime prevalence of insulin resistance and diabetes, cardiovascular disease, and higher BMI [58]. This study had limited First Nations involvement. A reduction in participation in research for minority groups is demonstrated in other areas of women’s health research [59]. Recent local research priority setting recognised First Nations health care as a research a priority area for our service [60]. Future studies should include consumers from the inception and development phases to optimise recruitment. This approach has been shown to improve research participation [61].

Although our study demonstrated favourable recruitment and completion rates across both the sleep study and randomisation to the CPAP intervention, overall recruitment numbers were low. Our results suggest high acceptability of both the sleep study and the intervention, but low overall recruitment, suggesting recruitment strategy should be reconsidered. Less than 10% of the eligible population were approached for participation in the study, and this is a significant limitation. Novel recruitment strategies such as the use of social media [48] and engagement with consumers to design recruitment may facilitate improved overall numbers in future studies [61]. Once engaged in the study, reasons for withdrawal were related to inconvenience and time availability rather than a lack of acceptability of the sleep study, highlighting the need to maximise research accessibility and convenience for women. Consideration of alternative diagnostic criteria for OSA [62], and the use of home-based noninvasive diagnostic technology [63], may increase recruitment rates by increasing convenience for participants. Overall, acceptability in the randomised group appeared high, with high completion rates and good device adherence overnight. This general acceptance of the intervention may arise from a selection bias created by the decision to only randomise women with moderate to severe (*AHI* ≥ 15) OSA. Severity of disease has previously been associated with better adherence to CPAP [64]. Broadening inclusion criteria for randomisation to include mild OSA (*AHI* > 5) may impact CPAP adherence in pregnancy.

With protocol modification, a multicentre trial is potentially feasible. We recommend future studies expand inclusion criteria and recruitment approaches. Future studies should examine minimum therapeutic efficacy in terms of timing and duration. Alternative methodologies to RCTs should be considered to allow examination of physiological effects mediated by intention and adherence to the device.

## Conclusion

In the Australian context, our feasibility study demonstrated low overall recruitment, suggesting that significant protocol modifications and resource allocation would be required to make a larger trial feasible. More robust screening techniques and broader inclusion criteria have the potential to increase overall recruitment. A diagnostic sleep study in pregnancy is an acceptable and feasible investigation. Testing the effectiveness and utility of CPAP in pregnancy via RCT appears to be both feasible and acceptable to women. Future studies should target clinical and cost-effectiveness including long-term paediatric outcomes and partial treatment effects. Adequate investigation of CPAP for the treatment of OSA in pregnancy necessitates adequate resources to meet the recruitment goals of intervention trials [65].

## Data Availability

The datasets during and/or analysed during the current study are available from the corresponding author on reasonable request.
